# Study protocol for *Women of Color and Asthma Control: *A randomized controlled trial of an asthma-management intervention for African American women

**DOI:** 10.1186/1471-2458-12-76

**Published:** 2012-01-24

**Authors:** Mary R Janevic, Georgiana M Sanders, Lara J Thomas, Darla M Williams, Belinda Nelson, Emma Gilchrist, Timothy RB Johnson, Noreen M Clark

**Affiliations:** 1 Center for Managing Chronic Disease, University of Michigan, 1415 Washington Heights, Ann Arbor, Michigan, USA; 2 University of Michigan Medical School and Departments of Internal Medicine and Pediatrics, 1500 E. Medical Center Drive, Ann Arbor, Michigan, USA; 3 University of Michigan Medical School and Department of Obstetrics and Gynecology, 1500 E. Medical Center Drive, Ann Arbor, Michigan, USA

**Keywords:** Asthma, randomized controlled trials, women, African Americans, chronic disease management, self-regulation, behavioral interventions

## Abstract

**Background:**

Among adults in the United States, asthma prevalence is disproportionately high among African American women; this group also experiences the highest levels of asthma-linked mortality and asthma-related health care utilization. Factors linked to biological sex (e.g., hormonal fluctuations), gender roles (e.g., exposure to certain triggers) and race (e.g., inadequate access to care) all contribute to the excess asthma burden in this group, and also shape the context within which African American women manage their condition. No prior interventions for improving asthma self-management have specifically targeted this vulnerable group of asthma patients. The current study aims to evaluate the efficacy of a culturally- and gender-relevant asthma-management intervention among African American women.

**Methods/Design:**

A randomized controlled trial will be used to compare a five-session asthma-management intervention with usual care. This intervention is delivered over the telephone by a trained health educator. Intervention content is informed by the principles of self-regulation for disease management, and all program activities and materials are designed to be responsive to the specific needs of African American women. We will recruit 420 female participants who self-identify as African American, and who have seen a clinician for persistent asthma in the last year. Half of these will receive the intervention. The primary outcomes, upon which the target sample size is based, are number of asthma-related emergency department visits and overnight hospitalizations in the last 12 months. We will also assess the effect of the intervention on asthma symptoms and asthma-related quality of life. Data will be collected via telephone survey and medical record review at baseline, and 12 and 24 months from baseline.

**Discussion:**

We seek to decrease asthma-related health care utilization and improve asthma-related quality of life in African American women with asthma, by offering them a culturally- and gender-relevant program to enhance asthma management. The results of this study will provide important information about the feasibility and value of this program in helping to address persistent racial and gender disparities in asthma outcomes.

**Trial Registration:**

ClinicalTrials.gov: NCT01117805

## Background

### Asthma and asthma-management among African-American women

Asthma prevalence in the United States continues to be at a worrying high level across the population, and both women and African Americans continue to be disproportionately affected by this illness. Among adults, prevalence among women is nearly double that among men (9.7 vs. 5.5%), representing a shift from childhood where asthma predominates among boys, and is higher among non-Hispanic Blacks compared to non-Hispanic Whites (8.7 vs. 8.1%) [1]. Gender and race disparities are also present in measures of asthma morbidity, emergency department use/hospitalization, and mortality [2-4].

The greater asthma incidence, prevalence and severity observed among women compared to men has been related to multiple issues; for example, hormonally-linked biological differences [5] and greater effects of smoking and obesity [5,6]. Certain risk factors for poor asthma outcomes such as depression and medication non-adherence may also be more common among women [6]. Similarly, multiple factors, many of which are associated with low socioeconomic status (SES), likely play a role in the greater asthma burden among African Americans; e.g., lack of access to optimal medical care, greater prevalence of indoor and outdoor environmental triggers commonly found in low-income areas, lack of asthma education and support, racial discrimination, and less use of anti-inflammatory medicines [7-10].

With excess risk conferred by both gender and race, African American women comprise a particularly vulnerable group of asthma patients, and are thus a logical focus of programs to improve asthma outcomes. The design of these programs should consider factors linked to both sex/gender and race that may affect asthma management. For example, many women experience menstruation-linked fluctuation in asthma symptoms and have greater exposure than men to common asthma triggers such as household cleaning products [11]. Compared to other ethnic groups, African American women have a higher average body mass index (BMI), which has been linked to more severe asthma symptoms [12]. In addition, among women screened for participation in a birth cohort study, African American women had higher levels of total IgE, with specific sensitivity to more aeroallergens, than their Caucasian counterparts [13]. They may also have a greater reliance on over-the-counter or home remedies and be more likely to delay care-seeking [14].

### Adapting *Women Breathe Free *for an African American population

A telephone-based asthma education intervention for women age 18 and over, *Women Breathe Free*, was the first published trial of an intervention addressing sex and gender-role issues in asthma management. This intervention was efficacious in reducing health care utilization while improving asthma-related quality of life and clinical status [15,16]. However, unpublished analyses examining the subgroup of African American women in this trial (n = 89) suggest that this group failed to achieve the same magnitude of positive outcomes as the overall study population, although statistical power was limited. These findings suggest the possibility that an intervention that better meets the specific asthma-education and support needs of African American women may achieve more positive results. There are few examples in the literature of asthma-management programs relevant to the needs of African American patients (see [17] for review), and none that are specific to women.

The *Women Breathe Free *program was adapted to meet the needs and preferences of African American women using a process that consisted of two primary phases: focus groups and expert review. In the first phase, forty-four African American women with asthma who had completed the *Women Breathe Free *study were recruited to take part in focus group discussions. Open-ended questions were posed about asthma-management concerns, and also the social, cultural, clinical, and practical aspects of an intervention that would help them better manage their asthma. Major themes that emerged from these groups included: difficulties associated with being overweight; extensive family care responsibilities; costs associated with asthma care and medicines as a barrier to management; the influence of social network members on asthma management; and the importance of social support. Desired characteristics of an asthma-education program included: a personalized program that addresses asthma-management challenges in the context of economic constraints; counselors who share the women's cultural background and demonstrate respect for their social and cultural experience, values, and beliefs; counselors who are knowledgeable, patient, and able to motivate and offer 'hand holding' when needed; delivered by telephone, a mode which the women found more flexible, personal, and private than group meetings; and materials that make use of checklists and graphics in place of extensive text.

After materials related to intervention content and process were initially developed based on focus group results, the second phase of adaptation, expert review, took place. A panel was convened consisting of three doctoral-level experts in asthma and cultural appropriateness (an epidemiologist/health educator, a cultural anthropologist, and a social worker). All panel members were themselves African American women who had extensive experience working in the field of asthma education in the African American community. This group reviewed all materials and made suggestions for modifications as needed. A summary of the ways in which the new program, called *Women of Color and Asthma Control (WCAC)*, is designed to be relevant to an African American population can be found in Table 1.

**Table 1:** Examples of culturally-relevant aspects of the *Women of Color and Asthma Control *intervention

Program element	Relevance
Program staff	Intervention counselors are African-American women, to enhance rapport with participants. Non-counseling staff are trained in issues related to cultural sensitivity.

Participant visuals	Photographs in workbook represent a diversity of African American women and families. Photos of telephone counselors included in workbook.

Workbook content	Use of culturally-relevant activities when discussing potential triggers (e.g., nail salons, church and family gatherings). Use of culturally-relevant examples in list of potential asthma-management problems (e.g., asthma medicine is expensive so I only buy it when my symptoms are bad, my family thinks prayer is all I need to take care of my asthma).

Telephone session content	Culturally-linked factors addressed by telephone counselors during program sessions, where relevant: --Potential perception that asthma is life-threatening and that fears, if not managed, can block effective action. --The role that being overweight may play in worsening asthma symptoms. --Impact of time and family responsibilities, including extended family. --Economic constraints in managing asthma. --The importance of community life and the potential influence on asthma management of the beliefs of other women in their social circles. --Potential use of alternative therapies. --Possibility of frequent asthma symptoms. --Possibility of use of urgent and emergency services for asthma as a regular feature of care. --Possibility of not using anti-inflammatory medicine. --Generation of a potential allergy profile to help in formulating strategies related to environmental control, potential seasons of increased risk, and the relationship between asthma and rhinitis. -Effective communication with health care team and other sources of support.

## Methods/Design

This study uses a randomized controlled design (intervention vs. usual care) to test the efficacy of *Women of Color and Asthma Control*, a telephone-based asthma management intervention for African American women. All study procedures have been reviewed and approved by the Institutional Review Board at the University of Michigan (IRB Study HUM00033784).

### Study hypotheses

Compared to women in the usual care group, women in the intervention group will: 1) use emergency department (ED) services for asthma less frequently and be hospitalized for asthma less frequently; 2) need urgent care in a physician's office less often; 3) experience fewer symptoms of asthma; and 4) have higher levels of asthma-related quality of life. We expect to see between-group differences at both 12 and 24 months from baseline.

### Sample size determination

The sample size of 420 at baseline (210 women in each treatment condition) was determined by power calculations using the primary study outcomes of ED visits and hospitalizations in the last 12 months, taking into account 30% attrition rate over 24 months and a loss of 5 women per group due to pregnancy. In previous work we observed that for ED visits and hospitalizations the treatment takes at least 12 months to reach full effect; thus, the sample size calculations for these outcomes were performed at 24 months. However, for the other outcomes, e.g. asthma symptoms and QOL, the intervention effect occurred during the first 12 months and the power calculations for these were performed at 12 months, which is the same or more conservative than 24 months.

The relatively rare occurrence of ED vists and hospitalizations for asthma requires a large sample size to detect clinical improvement over time. In previous work by the authors based on 364 participants with asthma, belonging to the intervention group was associated with a 75% reduction for hospitalization and 53% for ED visits with at least 80% power (alpha = 5%) at 24-month follow-up. We conservatively estimate a smaller reduction for the same two outcomes with a similar sample size and the same statistical power. An initial total sample of 420 and a final available sample of 286 will have sufficient power to detect a 32% improvement in the proportion of participants having at least 1 ED visit (42% in mean ED visits) and a 41% improvement in the proportion having at least one hospitalization (46% in mean hospitalizations). Note that these power calculations also ensure our ability to assess other important outcomes such as asthma symptoms and asthma quality of life.

### Participant recruitment

We are recruiting a total of 420 participants for this study over an approximately two-year period. To be eligible, participants must: 1) be a woman 18 years of age or older and self-identify as African American, 2) have access to a landline or mobile telephone, 3) not be pregnant, and 4) not reside in an institution.

Primary recruitment is through the University of Michigan Health System (UMHS), with eligibility requirements for participants including: 1) have at least one outpatient visit at the University of Michigan Health System (UMHS) in the past year; and 2) be listed on the UMHS Asthma Patient Registry, a validated all-payer registry of patients with persistent asthma cared for within UMHS. Lists of potential participants from the UMHS Asthma Registry are periodically generated and reviewed by a research nurse. Eligible patients are then mailed a packet including consent forms, a study overview page and a letter from the project director inviting them to enroll. The letter is followed by a recruitment phone call, which includes information about the study's purpose, randomization, and data collection. Upon returning a signed consent form, participants are enrolled in the study and randomized after baseline data collection.

Secondary recruitment methods include posting on the University of Michigan's clinical trial research database, http://UMClinicalStudies.org; partnering with the Blue Cross Blue Shield of Michigan (BCBSM) to identify members in the area who meet study criteria; and instituting a community recruitment plan that includes posting flyers in local health centers, hair salons, community organizations, and churches. All patients recruited outside of the Registry will have patient records in the UMHS or BCBSM systems that can be used for analysis of outcomes related to health care utilization. Any potential participant contacting study staff after learning about the study through one of these secondary recruitment methods will be screened for eligibility and then mailed the study packet as described above.

To enhance recruitment and retention of participants, a number of strategies are employed. Strategies include: 1) providing a $20 gift card upon completion of each data-collection interview; 2) mailing periodic greeting cards to establish a personal connection; 3) providing each intervention-group participant with her counselor's photograph and a personalized letter; 4) utilizing as recruiters, interviewers, and counselors individuals who share a cultural understanding and who have worked with and in the African American population; and 5) attempting contacts over a period of several weeks since disconnected phone lines are sometimes reconnected after a period of time.

### Randomization

After the baseline interview, participants are assigned to either treatment or control groups using a restricted randomization scheme. This scheme achieves balance between the two groups at all points during the study period. Prior to the beginning of the study, a permuted block randomization schedule for 420 subjects was created using the RANTBL and RANUNI functions in SAS to generate blocks with varying sizes of 8, 10, or 12 and random numbers for group assignment. After every block, there will be an equal number of intervention and control participants. This process ensures that over the course of the study, 210 women will be assigned to each of the two study conditions. It also reduces potential confounding effects (e.g., seasonal variation) which might occur due to the rolling recruitment process.

### Theory and content of intervention

*WCAC *is based on the Center for Managing Chronic Disease model of self-regulation for disease management, a framework that has been informed by social cognitive theory [18]. Participants are introduced to a self-regulatory problem-solving process, which is designed to help them engage more effectively in their asthma management. This process, which reflects the key components of the self-regulation model, include the following steps: 1) **Identifying a problem **in asthma management (participants are offered a list of potential asthma-management problems, including those associated with their sex, gender roles, culture and other relevant challenges; recommendations from the participant's physician are also reviewed at this stage); 2) **Observing **oneself, one's environment, and one's pattern of symptoms so as to understand both the influences on the problem and the way those influences might be ameliorated; 3) **Setting an asthma-management goal**; 4) **Developing a plan or strategy **to achieve the goal; and 5) **Tracking progress, reacting appropriately, and establishing suitable rewards **for success. Participants are guided by the counselor through a period of self-observation using a peak-flow meter and symptom diary, along with a checklist of physical activity, environmental factors, and other potential precipitants to asthma exacerbations. This observation period enables them to see the barriers and facilitators to achieving desired management practices and outcomes. Telephone sessions last 45 minutes to 1 hour and take place approximately every two weeks. Table 2 provides an overview of the content of each session. Importantly, the content of the intervention also reinforces the priority messages put forth by the National Asthma Education and Prevention Program's Guidelines Implementation Panel [19]: appropriate use of inhaled corticosteroids, reviewing asthma control, having an asthma action plan, periodic follow-up visits with clinician as appropriate, and allergen and irritant exposure control.

**Table 2:** *Women of Color and Asthma Control *intervention: Overview of program session content

Session	Examples of topics, and self-regulation phases addressed:	Goals for participant	Other content
1	Use of peak flow meter (PFM); tracking symptoms, medications and triggers; allergies.	Self-observe using asthma diary (mail copy of diary data back to health educator).	
	
2	Review PFM and symptom diary.	Use review to identify symptom patterns and triggers. Continue self-observation.	
	
3	Review PFM and symptom diary; review therapeutic plan provided by physician. Identify a management goal and develop plan to achieve goal.	Develop a plan for addressing the problem area and reaching goal. Carry out steps of the management plan.	Discussions of sex-, gender- and culture-related influences on asthma and asthma management are integrated into each session.
	
4	Review progress toward goal and adjust plan as needed.	Assess progress, fine-tune plan, and continue toward problem resolution.	
	
5	Review progress toward goal and consider next steps (e.g., refine plan, choose new problem, etc.). Discuss reward and benchmarks of progress.	Achieve goal as appropriate, apply problem solving process to new/different management problems.	

### Control condition

The control condition will consist of usual care from the UMHS or a BCBSM provider. At the UMHS, this care consists of evidence-based clinical practice and adherence to National Asthma Education and Prevention Program (NAEPP) guidelines for asthma diagnosis and treatment, and development of asthma action plans. Emphasis is also placed on patient education about disease processes, importance of trigger avoidance, and the development of a partnership for asthma care. Standardized asthma education kits are available in all UMHS clinic sites, and clinic personnel have been trained in their use by a certified asthma educator. Of note, sex-specific, gender-role, or culturally-related issues are not systematically or routinely addressed during the clinical encounter or in educational materials. All *WCAC *participants are sent basic asthma education materials ("Controlling Your Asthma" booklet from the American College of Chest Physicians) upon completion of the baseline interview.

### Outcomes and measures

Data is collected from participants via telephone interviews with trained research assistants. Data is collected on paper and then entered twice into Qualtrics, a secure online survey program, to ensure accuracy. With the participants' permission, an audio recording of the interview is also made to verify data accuracy and completion. Data collection takes place at three time points: baseline (before randomization), 12 months, and 24 months.

The primary study outcomes are the *number of asthma-related Emergency Department (ED) visits *and the *number of overnight hospitalizations *in the last 12 months. These will be collected via self-report on telephone interviews, as well as from medical and billing records.

Other outcomes of interest include the following:

#### Frequency of daytime and nighttime asthma symptoms

(coughing, wheezing, shortness of breath, and chest tightness), over the past month, seasonally, and in the last 12 months.

#### Asthma Control Test [20]

This validated, five-item questionnaire assesses the effect of asthma on daily functioning, shortness of breath, nighttime asthma symptoms, rescue medication use, and overall self-rated asthma control over the last 4 weeks.

#### Mini Asthma Quality of Life Questionnaire [21]

This validated 15-item scale addresses the following four domains specific to adult asthma patients: 1) activity limitations; 2) symptoms; 3) emotional function; and 4) environmental stimuli.

#### Absenteeism activity days

In the previous 12 months, 1) total days when physical activity was limited because of asthma; 2) total days of missed work or school because of asthma.

#### Other health care utilization

In the previous 12 months, unscheduled clinic visits for urgent asthma treatment and scheduled clinic visits for asthma care.

#### Asthma self-regulation skills and self-efficacy

A scale was developed by the authors that measures the frequency with which participants engage in the self-regulatory behaviors of observations, judgments, and reactions as applied to asthma management; and also measures self-efficacy for each of these behaviors.

#### Sex/gender-specific management problems

This scale, used in previous work by the authors [15], measures the frequency of problems of asthma management related to hormonal cycles, sexual activity, urinary incontinence, and triggers associated with gender roles.

#### African-American-specific management problems

This scale, developed by the authors, measures the frequency with which participants experience asthma-management challenges that may be common among African American women (e.g., having numerous family responsibilities, physician communication, money concerns or worries).

Data is also collected on a wide array of demographic, health and psychosocial characteristics at all three time points.

### Analysis

Statistical testing will evaluate changes in the primary and secondary outcomes, and other outcomes of interest, over the course of the study, and assess differences in these changes as a function of participation in the intervention. Descriptive statistics will be computed for all outcomes, as well as sociodemographic and health characteristics, overall and for each study group (treatment and control). This will help determine whether the treatment and usual-care groups are equivalent prior to intervention, and how these groups differ at specific follow-up points (12 and 24 months after baseline). We will use mixed-effects models for the analysis of continuous outcome variables and generalized estimating equations (GEEs) for analyzing discrete outcomes. Pre-planned contrasts will examine the nature of change over time, including the timing and longevity of intervention effects. SAS Version 9.2 and IVEware (to multiply impute missing data) will be used for analysis [22,23].

### Process evaluation

Data will also be collected regarding key aspects of the intervention process. First, participants will be asked to complete a brief online or mail survey regarding their experience with the WCAC program (e.g., satisfaction with counselor, perceived improvement in asthma-management skills) when they have completed the series of telephone counseling sessions. This survey also includes items designed to assess participants' satisfaction with the culturally-relevant elements of the program (e.g., "If you were to participate in a program like this one again, how important would it be to you that your health educator be African American?"). Second, telephone counselors will keep logs of each phone session to track elements such as participants' goals and progress in the self-regulation process. Finally, all telephone counseling sessions are audio-recorded and a random subset are reviewed for their fidelity to the planned program content. This process data will be used to enhance understanding of program outcomes, and to inform modifications to future versions of the program.

## Discussion

While African American women with asthma are at higher risk than their Caucasian counterparts of poor asthma-related outcomes, no prior asthma-management intervention has been adapted to address their particular needs and cultural context. The current trial will answer the question: Does a program relevant to the gender and culture of the participants reduce the burden of asthma on a particularly vulnerable group? To do this, it will test the efficacy of the WCAC program in reducing asthma-related ED use, hospitalizations, and symptoms, as well as improving asthma-related quality of life. It will also provide important information about the receptiveness of participants to an intervention relevant in this manner. If shown to be efficacious, the WCAC program will represent one means of addressing persistent racial disparities in asthma outcomes.

## List of abbreviations used

ED: Emergency Department; WCAC: Women of Color and Asthma Control; PFM: peak flow meter; QOL: quality of life; SES: socioeconomic status; BMI: body mass index; UMHS: University of Michigan Health System; BCBSM: Blue Cross Blue Shield of Michigan.

## Competing interests

The authors declare that they have no competing interests.

## Authors' contributions

NMC is the Principal Investigator of the study, and GMS and TRBJ are Co-investigators; MRJ is a Research Associate with the study. LJT, DMW, BN, and EG are involved in project management and/or intervention delivery. All authors participated in the design of the trial, intervention, and/or measures. MRJ and NMC drafted the manuscript; all authors reviewed, edited, and approved the final manuscript.

## Pre-publication history

The pre-publication history for this paper can be accessed here:

http://www.biomedcentral.com/1471-2458/12/76/prepub

## References

[B1] Centers for Disease Control and Prevention (CDC) Vital signs: asthma prevalence, disease characteristics, and self-management education: United States, 2001--2009 MMWR Morb Mortal Wkly Rep 2011 60 17 547 552 21544044

[B2] Kynyk JA Mastronarde JG McCallister JW Asthma, the sex difference Curr Opin Pulm Med 2011 17 1 6 11 10.1097/MCP.0b013e3283410038 21045697

[B3] Moorman JE Mannino DM Increasing U.S. asthma mortality rates: who is really dying? J Asthma 2001 38 1 65 71 10.1081/JAS-100000023 11256556

[B4] Zoratti EM Havstad S Rodriguez J Robens-Paradise Y Lafata JE McCarthy B Health service use by African Americans and Caucasians with asthma in a managed care setting Am J Respir Crit Care Med 1998 158 2 371 377 9700109 10.1164/ajrccm.158.2.9608039

[B5] Melgert BN Ray A Hylkema MN Timens W Postma DS Are there reasons why adult asthma is more common in females? Curr Allergy Asthma Rep 2007 7 2 143 150 10.1007/s11882-007-0012-4 17437685

[B6] Ostrom NK Women with asthma: a review of potential variables and preferred medical management Ann Allergy Asthma Immunol 2006 96 5 655 665 10.1016/S1081-1206(10)61062-9 16729777

[B7] Krieger JW Takaro TK Song L Weaver M The Seattle-King County Healthy Homes Project: a randomized, controlled trial of a community health worker intervention to decrease exposure to indoor asthma triggers Am J Public Health 2005 95 4 652 659 10.2105/AJPH.2004.042994 15798126 PMC1449237

[B8] Wright RJ Rodriguez M Cohen S Review of psychosocial stress and asthma: an integrated biopsychosocial approach Thorax 1998 53 12 1066 1074 10.1136/thx.53.12.1066 10195081 PMC1745142

[B9] Fuhlbrigge AL Adams RJ Guilbert TW Grant E Lozano P Janson SL Martinez F Weiss KB Weiss ST The burden of asthma in the United States: level and distribution are dependent on interpretation of the National Asthma Education and Prevention Program guidelines Am J Respir Crit Care Med 2002 166 8 1044 1049 10.1164/rccm.2107057 12379546

[B10] Adams RJ Weiss ST Fuhlbrigge A How and by whom care is delivered influences anti-inflammatory use in asthma: Results of a national population survey J Allergy Clin Immunol 2003 112 2 445 450 10.1067/mai.2003.1625 12897755

[B11] Clark NM Valerio MA Gong ZM Self-regulation and women with asthma Curr Opin Allergy Clin Immunol 2008 8 3 222 227 10.1097/ACI.0b013e3282fe9d05 18560296 PMC3143000

[B12] Beuther DA Obesity and asthma Clin Chest Med 2009 30 3 479 88 viii 10.1016/j.ccm.2009.05.002 19700046

[B13] Litonjua AA Celedon JC Hausmann J Nikolov M Sredl D Ryan L Platts-Mills TA Weiss ST Gold DR Variation in total and specific IgE: effects of ethnicity and socioeconomic status J Allergy Clin Immunol 2005 115 4 751 757 10.1016/j.jaci.2004.12.1138 15805994

[B14] American College of Chest Physicians Communicating With Asthma Patients of Diverse Cultures 2006 http://www.chestfoundation.org/downloads/communityResources/Asthma06.pdf

[B15] Clark NM Gong ZM Wang SJ Valerio MA Bria WF Johnson TR From the female perspective: Long-term effects on quality of life of a program for women with asthma Gend Med 2010 7 2 125 136 10.1016/j.genm.2010.04.005 20435275 PMC3146346

[B16] Clark NM Gong ZM Wang SJ Lin X Bria WF Johnson TR A randomized trial of a self-regulation intervention for women with asthma Chest 2007 132 1 88 97 10.1378/chest.06-2539 17505047

[B17] Bailey EJ Cates CJ Kruske SG Morris PS Brown N Chang AB Culture-specific programs for children and adults from minority groups who have asthma Cochrane Database Syst Rev 2009 2 April 15 CD006580 18425956 10.1002/14651858.CD006580.pub2

[B18] Clark NM Gong M Kaciroti N A model of self-regulation for control of chronic disease Health Educ Behav 2001 28 6 769 782 10.1177/109019810102800608 11720277

[B19] U.S. Department of Health and Human Services, National Institutes of Health, National Heart, Lung, and Blood Institute National Asthma Education and Prevention Program Expert Panel Report 3: Guidelines for the Diagnosis and Management of Asthma 2007 NIH Publication No. 07-4051

[B20] Nathan RA Sorkness CA Kosinski M Schatz M Li JT Marcus P Murray JJ Pendergraft TB Development of the Asthma Control Test: A survey for assessing asthma control J Allergy Clin Immunol 2004 113 1 59 65 10.1016/j.jaci.2003.09.008 14713908

[B21] Juniper EF O'Byrne PM Guyatt GH Ferrie PJ King DR Development and validation of a questionnaire to measure asthma control Eur Respir J 1999 14 4 902 907 10.1034/j.1399-3003.1999.14d29.x 10573240

[B22] SAS Institute Inc. SAS Version 9.2. [Computer software]. Cary, N.C. USA 2008

[B23] Raghunathan TE Solenberger PW Van Hoewyk J IVEware: Imputation and variance estimation software [Computer software] 2002

